# Extruded Wheat Bran Consumption Increases Serum Short-Chain Fatty Acids but Does Not Modulate Psychobiological Functions in Healthy Men: A Randomized, Placebo-Controlled Trial

**DOI:** 10.3389/fnut.2022.896154

**Published:** 2022-05-26

**Authors:** Boushra Dalile, Danique La Torre, Polona Kalc, Francesca Zoppas, Chiara Roye, Chrystel Loret, Lisa Lamothe, Gabriela Bergonzelli, Christophe M. Courtin, Bram Vervliet, Lukas Van Oudenhove, Kristin Verbeke

**Affiliations:** ^1^Translational Research Center in Gastrointestinal Disorders (TARGID), Department of Chronic Diseases and Metabolism, Faculty of Medicine, KU Leuven, Leuven, Belgium; ^2^Leuven Brain Institute, KU Leuven, Leuven, Belgium; ^3^Laboratory of Food Chemistry and Biochemistry and Leuven Food Science and Nutrition Research Centre (LFoRCe), KU Leuven, Leuven, Belgium; ^4^Nestlé Product Technology Centre, Coffee Société des Produits Nestlé S.A., Lausanne, Switzerland; ^5^Nestlé Institute of Materials Science, Nestlé Research, Société des Produits Nestlé S.A., Lausanne, Switzerland; ^6^Nestlé Institute of Health Sciences, Nestlé Research, Société des Produits Nestlé S.A., Lausanne, Switzerland; ^7^Laboratory of Biological Psychology, Brain & Cognition, Faculty of Psychology and Educational Sciences, KU Leuven, Leuven, Belgium; ^8^Cognitive and Affective Neuroscience Lab, Department of Psychological and Brain Sciences, Dartmouth College, Hanover, NH, United States

**Keywords:** short-chain fatty acids, psychosocial stress, fear conditioning, wheat bran, dietary fiber, gut-brain axis

## Abstract

**Background:**

Incorporation of wheat bran (WB) into food products increases intake of dietary fiber, which has been associated with improved mood and cognition and a lower risk for psychiatric disorders such as depression, with short-chain fatty acids (SCFAs) as candidate mediators of these effects. Modifying WB using extrusion cooking increases SCFA production *in vitro* relative to unmodified WB.

**Objective:**

The aim of this study was to evaluate the effects of extruded WB on psychobiological functioning and the mediating role of SCFAs.

**Methods:**

In a randomized, triple-blind, placebo-controlled trial, 69 healthy male participants consumed 55 g of breakfast cereal containing either extruded WB or placebo daily for 28 days. At pre- and post-intervention visits, the cortisol response to experimentally induced stress was measured as a primary outcome. In addition, serum SCFAs and brain-derived neurotrophic factors were quantified as potential mediators. Secondary psychobiological outcomes included subjective stress responses, responses to experimentally induced fear, cortisol awakening response, heart rate variability, and retrospective subjective mood ratings. Intestinal permeability, fecal SCFAs, and stool consistency were measured as secondary biological outcomes.

**Results:**

Extruded WB increased serum acetate and butyrate (*p* < 0.05). None of the primary or secondary outcomes were affected by the intervention. Participants who consumed a placebo exhibited an increase in the percentage of fecal dry weight but did not report increased constipation. Despite these statistically significant effects, these changes were small in magnitude.

**Conclusions:**

Extruded WB consumption increased serum short-chain fatty acids but did not modulate psychobiological functions in healthy men. Effective modulation of psychobiological functions may require greater increases in SCFAs than those achieved following extruded WB consumption. Rather than attempting to induce health benefits with a single fiber-rich food, combinations of different fibers, particularly highly fermentable ones, might be needed to further increase SCFA production and uptake in the systemic circulation to observe an effect on psychobiological processes.

## Introduction

Dietary fiber comprises different types of carbohydrate polymers that cannot be digested nor absorbed by the human small intestine and is predominantly found in fruits, vegetables, legumes, and cereals. Low dietary fiber intake is not only associated with coronary heart disease, stroke, hypertension, diabetes, obesity, and certain gastrointestinal diseases (1) but also with worsened cognition and mood and the development of mental health disorders (2–5). It is often hypothesized that the fermentation of dietary fiber by the resident gut bacteria and the subsequent production of short-chain fatty acids (SCFAs) in the colon drive the effects of dietary fiber on mental health via the microbiota-gut-brain axis (6). SCFAs function as primary energy sources for luminal colon cells. Besides exerting local effects in the colon, including maintenance of gut barrier integrity, anti-inflammatory, and anticarcinogenic effects, SCFAs can mediate microbial signaling to the brain via multiple pathways (6). SCFAs affect gene expression by inhibiting histone deacetylases (HDACs) and act as endogenous ligands for orphan G-protein coupled receptors. In addition, SCFAs modulate systemic inflammatory and gastrointestinal endocrine responses, cross the blood-brain barrier, and signal to the brain via vagal afferents, thereby interacting with virtually all systems mediating gut-brain communication [reviewed extensively elsewhere (6)]. In a previous study, we provided a proof of concept that SCFAs affect the hypothalamic-pituitary-adrenal axis – the main physiological system that regulates the body's response to stress via the release of endocrine hormones such as cortisol, and that SCFAs are dysregulated in various diseases (7). Specifically, daily administration of known amounts of an SCFA mixture to the colon of healthy men for one week significantly reduced the cortisol response to an acute psychosocial stress challenge (8). Furthermore, the increase in circulating SCFAs was associated with a decrease in cortisol response to stress (8). While the mechanisms through which SCFAs modulate the HPA axis response to stress are currently unclear, these findings suggest that SCFAs are an important mechanism through which fermentation of dietary fiber could regulate the HPA axis reactivity to stress and may play a role in the development of mental disorders (9).

While most countries recommend a daily intake of dietary fiber of 25–32 g/day for adult women and 30–35 g/day for adult men, average intakes fall behind (10). In Europe, average intakes ranged from 18 to 24 g/day for adult men and from 16 to 20 g/day for women, with little variation from one European country to another. The lowest figures are recorded for Canada and the USA (10). Therefore, incorporating food products into the diet to increase daily fiber intake is essential and may mitigate the development of a myriad of physical and mental health issues, potentially via increased SCFA production. To begin addressing this hypothesis, we examined the effects of a rich, abundant, and cheap source of dietary fiber – wheat (*Triticum aestivum L*.) bran (WB) – on psychobiological processes in healthy participants and the mediating role of SCFAs herein.

Wheat bran comprises the outer layer of the wheat kernel, including the pericarp, testa, and aleurone layer. About 6.5 million tons of WB is produced yearly as a byproduct of the flour milling industry in Europe (11) and is mostly used in animal feed as a source of phosphorus and protein, to lighten dense and heavy feed mixtures and to increase fiber intake in animals (12). WB is a concentrated source of dietary fiber (43–62%) and it further contains starch (6–19%), protein (14–18%), lipids (3–6%), and ash (5– 8%) (11). The dietary fiber fraction of WB mainly consists of insoluble dietary fiber such as water-unextractable arabinoxylan, water-unextractable β-glucan, and cellulose. This fraction binds water in the gastrointestinal tract, increases fecal bulk, and normalizes transit time (13–15). Moreover, the insoluble polymers can be seen as a nutrient platform for the gut microbiota, improving the efficiency of microbial fermentation (16, 17). Only a small fraction (4 to 10%) of the total dietary fiber fraction of WB is soluble (i.e., water-extractable arabinoxylan, water-extractable β-glucan, and fructan) (18). The soluble dietary fiber is fermented by the gut microbiota, which results in the production of SCFAs. SCFAs are hypothesized to be one of the putative mechanisms for various health effects of WB (11), such as the prevention of colorectal cancer, cardiovascular disease, obesity, and some gastrointestinal symptoms and disorders, including constipation and irritable bowel syndrome (IBS) (19).

Despite the abundance of WB, its high fiber content, its potential health benefits, and its incorporation into food products such as bread (18) and cookies (20) negatively impact their organoleptic quality, namely impacting texture and decreasing sensory acceptance. Moreover, the rigid structure of WB limits the availability of potential nutrients. Modifications of the physical properties of WB or fractionating WB to obtain fractions enriched in tissues of interest can improve its techno-functional properties but may also increase its colonic fermentability and thereby modulate health outcomes (11). One such modification method is extrusion-cooking, a technique that combines high temperature, high pressure, and high shear. We previously demonstrated that extrusion-cooking degraded aleurone cells, increased soluble dietary fiber levels, improved the water-binding capacity, degraded phytate, and released ferulic acid. In addition, extrusion-cooking increased the fermentation rate and degree during *in vitro* fermentation (21, 22). Building on this previous study, the current work examined the effects of extrusion-cooked WB on psychobiological outcomes in a sample of healthy men in a randomized triple-blind, placebo-controlled intervention and quantified circulating SCFA concentrations as a potential mediator.

## Materials and Methods

### Subjects

For this study, 81 men between the ages of 20 and 40 years and with a normal BMI (18.5–25 kg/m^2^) were recruited. Women were excluded for previously cited reasons (8). The exclusion criteria included having previous or current neurological, psychiatric, gastrointestinal, or endocrine disorders; substance/alcohol dependence or abuse (>2 units per day/14 units per week); having current or recent regular medication use; having one or more diagnoses based on the MINI-international neuropsychiatric interview; having one or more diagnoses based on ROME IV criteria for functional GI disorders; being in night-shift work; adherence to vegan, vegetarian, or special diets; smoking; using prebiotics or probiotics within 1 month preceding the study; using antibiotics within 3 months preceding the study; daily fiber consumption higher than 25 g; and having previous experience with experimental psychological tasks. All participants gave written consent to participate and were compensated for their time. This clinical trial was approved by the Medical Ethics Committee of UZ Leuven/KU Leuven (S62344), conducted in accordance with the Declaration of Helsinki, and pre-registered on ClinicalTrials.gov (Identifier: NCT04522258). The data were collected in the StressLab, located at the Psychiatry division of the UZ Leuven, and took place between May 2019 and November 2020.

### Experimental Protocol

**Figure 1:**
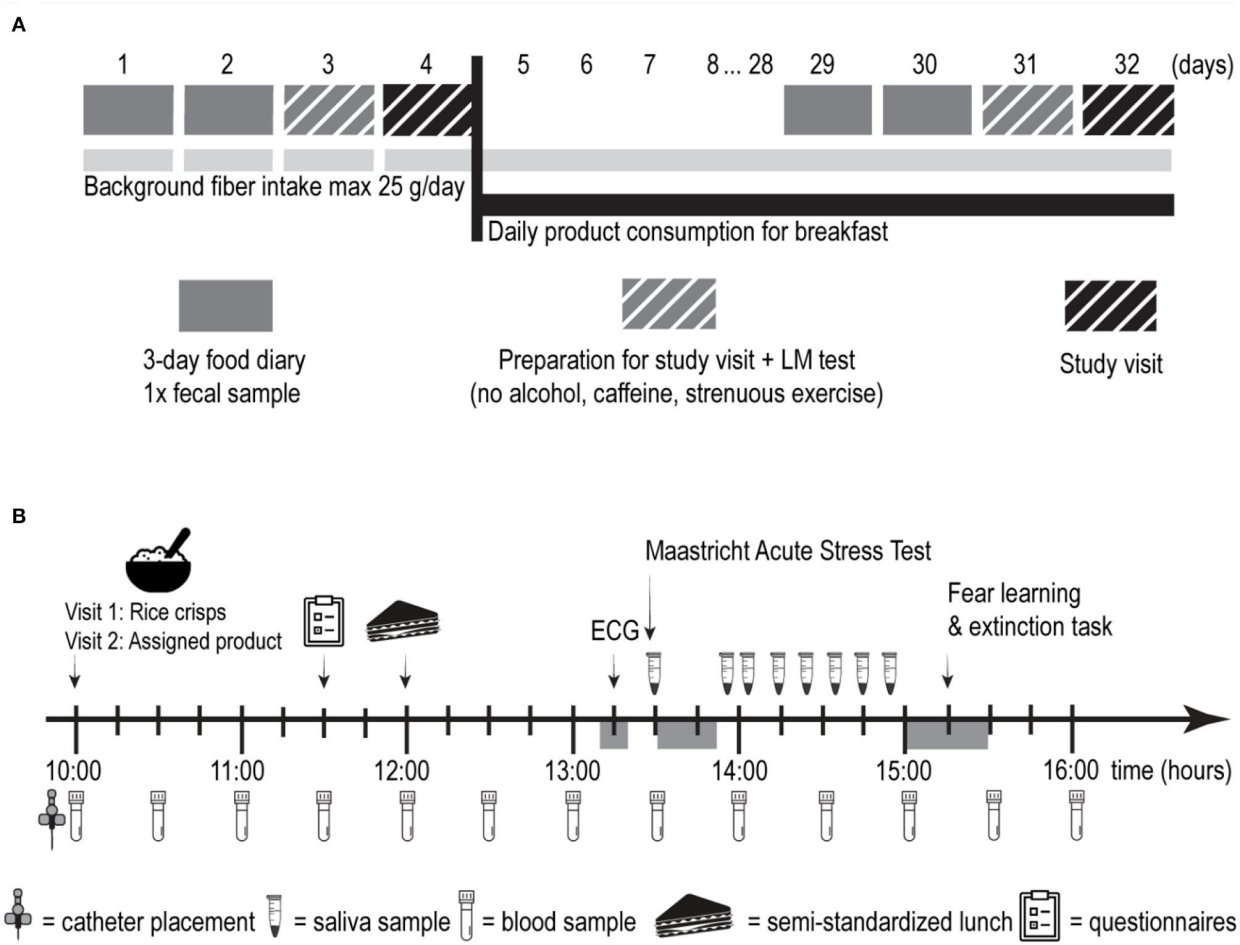
Overview of the experimental protocol. **(A)** Overview of the study design. **(B)** Schematic overview of one study visit. LM, lactulose/mannitol; ECG, electrocardiography.

A four-week, randomized, triple-blind, placebo-controlled trial was performed in a parallel-group design. Power calculation and randomization protocol are reported as Supplementary Material. One group received one portion of an extruded breakfast cereal based on WB whereas the other group received an extruded breakfast cereal based on wheat flour and the inert dietary fiber microcrystalline cellulose (50:50 w/w%; placebo). The trial comprised two study visits (Figure 1A). Visit 1 was a baseline visit (pre-intervention) and visit 2 was scheduled following a 28-day intervention with extruded WB or placebo (post-intervention). During the 3 days prior to each study visit, participants registered food intake electronically using www.myfitnesspal.com (Supplementary Material) and collected one fecal sample for quantification of fecal SCFA concentrations. On the day prior to each study visit, they performed a standard differential urinary sugar excretion test ([actulose/mannitol (LM)] at home, and they were asked to refrain from the consumption of caffeine and alcohol or performing strenuous physical exercise. Participants were requested to keep their fiber intake under 25 g per day throughout the study period.

Each study visit (Figure 1B) began with the collection of saliva samples at home, before participants' arrival at the lab in a fasted state. Saliva samples were later used to quantify the cortisol awakening response (CAR). After placement of an I.V. catheter and collection of a baseline serum sample, they consumed a standardized no-fiber breakfast on the pre-intervention visit and the test breakfast (placebo or extruded WB) on the post-intervention visit. Then, 1 h and 45 min later, participants completed a battery of questionnaires and consumed a standardized no-fiber lunch *ad libitum* afterward. Details of the standardized breakfast and lunch can be found in Supplementary Material. Then, 1 h after lunch, participants' electrocardiographic activity was measured to quantify resting heart rate variability (HRV). At after 15 min, participants underwent the stress induction procedure and collected saliva samples for 1 h. This was followed by a computerized fear learning and extinction task, in which acquisition and extinction of fear occurred at the pre-intervention visit, and the return of fear was tested at the post-intervention visit. Blood samples were collected upon arrival at the lab (T+0h00) and every 30 min thereafter for 5 h and 30 min, leading to a total of 12 blood samples. As we were interested in how postprandial SCFA concentrations change in response to the intervention, all serum samples were analyzed for SCFAs. SCFAs have a short half-life, rendering information from only a fasting sample less relevant. Brain-derived neurotrophic factor (BDNF) was only quantified in the fasting serum sample in accordance with the previous literature (23), and since previous study showed a relationship between fiber intake and fasting BDNF concentrations (24) but not between various macronutrient intake and postprandial BDNF concentrations (25).”

### Intervention Products

#### Production of a Wheat Bran-Based Breakfast Cereal

Wheat bran was purchased from Dossche Mills (Deinze, Belgium) and contained 11.8% starch, 20.9% proteins, 5.4% lipids, 24.4% water-unextractable AX, 0.6% water-extractable AX, 9.1% cellulose, 2.1% (1,3:1,4)-β-glucan, 3.3% fructan, 4.3% phytate, 11% lignin, and 6.4% ash on dry matter (dm) basis (17). The total dietary fiber content was calculated as the sum of arabinoxylan, cellulose, β-glucan, fructan, and lignin and was 50.5% dm. The average particle size of wheat bran was 1,420 μm. Avicel GP 1,030 cellulose was purchased at Dupont (Cork, Ireland). The degree of polymerization of the cellulose was not more than 350, and the crystallinity was approximately 82%.

Native WB was extruded using a BC72 industrial-scale extruder (Clextral, Firmini, France), resulting in the production of an extruded breakfast cereal consisting of 50.5% dm of dietary fiber. System parameters, i.e., specific mechanical energy, product temperature, and pressure in the last barrel were measured as detailed previously (22) and are given in Supplementary Table S1. The samples exiting the extruder were cut into strands of approximately 1 cm and were dried. Samples were then coated with a 5% sucrose solution and dried again. The samples were packed in 55-g portions corresponding to 25 g of dietary fiber per package.

#### Production of the Placebo Breakfast Cereal

The placebo sample was produced by extruding 50% wheat flour and 50% microcrystalline cellulose (MCC) using the BC72 extruder (Clextral, Firmini, France). Wheat flour was purchased at Dossche Mills (Deinze, Belgium). This recipe was chosen to equalize the amount of dietary fiber in the wheat bran-based breakfast cereal and the placebo product (both 50% dm). MCC is an inert fiber that is hardly fermentable. Wheat flour serves as filling material and mainly consists of starch and proteins, which are digested and absorbed in the small intestine, do not reach the colon, and do not interfere in the experimental set-up. The product was colored using 2% of caramel coloring to look as similar as possible to the active product. Afterward, the product was coated with 5% sucrose solution, dried, and packed in 55-g portions corresponding to 25 g of dietary fiber per package.

#### Characterization of the Extruded Samples

The product that exited the extruder (before applying the sugar coating) was milled and used for characterization. The strong water-binding capacity of the samples (17), total water retention capacity (26), swelling capacity (27), and extractability on the bran (17) were determined.

### Psychological Readouts

#### Cortisol Awakening Response

The salivary cortisol awakening response (CAR) (28) was assessed by instructing participants to provide five saliva samples using the synthetic Salivette Cortisol (Sarstedt AG & Co., Nümbrecht, Germany) immediately upon waking up and subsequently every 15 min.

#### Questionnaires

Participants completed a battery of questionnaires during each study visit (see Supplementary Material for details). The Positive and Negative Affect Schedule (PANAS) (29) assessed positive and negative emotional states. The Perceived Stress Scale (PSS) (30) assessed the degree to which situations in one's life are appraised as stressful. The Depression, Anxiety, Stress Scales (DASS-21) (31) assessed multiple dimensions of these mood states, including but not limited to dysphoria, hopelessness, situational anxiety, and levels of chronic non-specific arousal related to stress. The Leiden Index of Depression Sensitivity-Revised (LEIDS-R) (32) assessed cognitive reactivity to sad moods. Finally, participants also completed the Gastrointestinal Symptom Rating Scale (GSRS) (33), which assessed gastrointestinal symptoms including reflux, abdominal pain, indigestion, diarrhea, and constipation. Participants rated their symptoms and mood with “over the past month” as a reference.

#### Heart Rate Variability (HRV)

The ECG was obtained using three standard Ag/AgCl electrodes (1 cm diameter) filled with electrolyte and placed on the thorax across the heart: two electrodes were placed below the left and right clavicles and one electrode was placed on the left lower rib cage. The signal was sampled at 1,000 Hz and transduced, amplified, and filtered through a Coulbourn S75-04 Isolated Bioamplifier (Coulbourn Instruments, Lehigh Valley, Pennsylvania). Low frequencies were cut off at 10 Hz and high frequencies at 1 kHz. The signal was visually inspected and artifacts were corrected. Interbeat intervals were extracted from the filtered signal, from which the root mean square of the successive differences (RMSSD) between heart rates was calculated as an index of HRV using Kubios HRV (version 2, BSAMIG, Finland).

#### Stress Sensitivity

The Maastricht Acute Stress Test (MAST) (34) consists of a 5-min instruction phase and a 10-min acute stress phase. The acute stress phase comprises multiple trials in which participants immerse their hands in the cold (2°C) water for a period not exceeding 90 s. In between the hand-immersion trials, participants performed a mental arithmetic task, counting backward from 2,043 in steps of 17 or 13 (counterbalanced across the groups and study visits) as quickly and as accurately as possible for at least 45 s before the next hand-immersion trial starts. To induce social stress, participants were monitored by a woman experimenter dressed in a white lab coat, were given negative feedback upon making a mistake, and were “mock” videotaped throughout the task to later analyze their facial expressions. This task elicits a robust subjective and physiological stress response (34). Moreover, no significant habituation or sensitization to the MAST was found upon repeated administration (35). To measure cortisol, saliva samples were obtained with synthetic Salivette (Sarstedt AG & Co., Nümbrecht, Germany) 5 min before (i.e., *t*_pre−stress(−20min)_) and 7 times after stress induction (i.e., *t*_+00_, *t*_+05_, *t*_+15_, *t*_+25_, *t*_+35_, *t*_+45_, and *t*_+55_ min). To measure subjective stress response, participants rated how stressful, painful, and unpleasant they felt using visual analog scales (VAS) (anchors: 0 = “*not at all”*; 10 = “*extremely”*) in the middle and at the end of the MAST compared to baseline values.

#### Fear Learning and Extinction Task

A cue- and context-dependent Pavlovian fear learning and extinction paradigm was administered to investigate the effect of the extruded WB vs. placebo consumption on learning and memory processes involved in anxiety disorders (36). In this paradigm, a previously neutral conditional stimulus (CS+, e.g., blue light,) predicted an unconditional stimulus (US): a mild electrical stimulation in a given context (in context A but not in context B) while another neutral conditional stimulus (e.g., yellow light, CS–) never predicted electrical stimulation in either context. This paradigm was used to induce and extinguish fear in otherwise healthy participants at the pre-intervention visit and measure the return of fear after a lapse of time (recall), after the presentation of an unsignaled US to reinstate the original fear response (reinstatement) or after the contextual change (renewal) at the post-intervention visit. Fear responses were assessed by analyzing skin conductance responses (SCRs) and US expectancy ratings (see Supplementary Material for details).

### Biological Readouts

#### Serum SCFAs

Blood samples were collected into a red top glass serum tube (37). Twelve blood samples were collected at 30-min intervals during each study visit, with the first one collected in a fasted state upon arrival at the lab. The serum was then separated and stored at −80°C until analysis. Total SCFA concentrations were measured using gas chromatography-mass spectrometry (GC-MS) after derivatization with 2,4-difluoroanilin and subsequent extraction into ethyl acetate as described previously (38) with optimization of the derivatization method.

#### Fecal SCFAs and Fecal Dry Weight

Fecal samples were collected within 3 days before the pre- and post-intervention visits and used to measure the excreted SCFA concentrations. Samples were collected at home and stored in the freezer. They were transported in a frozen state by the participants on each study visit and immediately stored at −80°C until analysis. The SCFAs were extracted from the samples with diethyl ether and quantified using gas chromatography with flame-ionization detection (GC-FID) (39). The percentage of fecal dry weight was calculated by dividing the weight of the lyophilized sample by the wet fecal sample and multiplying it by 100.

#### Cortisol Analysis

Saliva samples for the CAR and the MAST were collected and stored at −20°C until analysis. The samples were analyzed using Salivary Cortisol ELISA SLV-2930 (DRG Instruments GmbH, Marburg, Germany) according to the manufacturer's instructions.

#### BDNF Analysis

Serum BDNF concentrations were quantified in the fasted serum sample using a quantitative sandwich enzyme immunoassay technique using the human BDNF ELISA kit (Biosensis, Calbiotech Inc., USA), according to the manufacturer's instructions (23).

#### Intestinal Permeability

*In vivo* intestinal permeability was measured using a standard differential urinary sugar excretion test (40, 41). Participants were requested to void their bladder after an overnight fast and to ingest a test solution that contains 5 g lactulose (Eurogenerics, Brussels, Belgium) and 1 g mannitol (ABC Chemicals, Mississauga, Ontario, Canada) in 150 ml water. Then, 45 min after drinking the test solution, subjects drank 250 ml of water to stimulate urine production. Urine was collected for 2 h in a pre-weighed plastic container with 250 mg of neomycin to prevent bacterial degradation of lactulose and mannitol. After collection, the total urine volume was noted and 1.5 ml sample aliquots were filtered with 0.45 μm filters (Merck Millipore, Billerica, Massachusetts, USA) and stored at −20°C until further analysis. Urinary lactulose and mannitol concentrations were measured with HPLC -ELSD as described previously (42). The lactulose-mannitol ratio (LMR) was used as a marker for small intestinal permeability (40, 43, 44). In addition, the fractional excretion of lactulose (FEL) and mannitol (FEM) were calculated as done previously (45).

### Statistical Analysis

Statistical analysis was conducted using *SAS*® software version 9.4 (SAS Institute, Cary, NC, USA). The primary outcome of interest was the cortisol response to stress. Secondary outcomes were psychophysiological responses (fear learning and extinction, cortisol awakening response, HRV, and self-report questionnaires) and biological measures (intestinal permeability and fecal SCFAs). Serum SCFAs and BDNF levels were measured as potential mediators. Data derived from the stress procedure (cortisol and VAS), the fear learning and extinction procedure (US expectancy ratings and SCRs), and biological samples (CAR, serum and fecal SCFAs, serum BDNF, and small intestinal permeability) were analyzed using linear mixed models (after appropriate transformation of the dependent variables in case of non-Gaussian distribution) with “group” (extruded WB vs. placebo) as between-subject factor and “visit” (pre- and post-intervention) and, where appropriate, “timepoint” or “phase” (measurement point within each visit) as within-subject factors, as done previously (46). This procedure prevented listwise deletion due to missing data. Furthermore, the covariance between test visits is accounted for in our model by choosing an unstructured covariance matrix for the visited variable, allowing it to differ between groups. The group-by-visit and the group-by-visit-by-timepoint/phase interaction effects constituted the principal effects of interest. For group-by-visit-by-timepoint/phase interaction, follow-up planned contrasts were conducted following significant interaction effects (*p*-value <0.05) to test differences between extruded WB vs. placebo groups per timepoint. Stepdown Bonferroni (Holm) adjustment was used to correct for multiple testing comparisons. Questionnaire data that did not follow Gaussian distribution and could not be transformed were divided into tertiles or quartiles and were analyzed using generalized linear mixed models with a cumulative logit link function for ordinal response variables.

## Results

### Participant Characteristics

Among the 81 participants who were recruited for the study, 12 did not complete the intervention period for various reasons (*n* = 2 dropped out for undisclosed reasons, *n* = 1 reported non-compliance with the study protocol prior to the post-intervention visit, and *n* = 9 dropped out due to COVID-19 related issues). The final sample comprised 69 healthy male participants (*M*_age_ = 25.98, *SD*_age_ = 4.09; *M*_BMI_ = 22.88, *SD*_BMI_ = 1.81), of which 34 received extruded WB and 35 received placebo.

### Characterization of the Extruded Products and Analysis of Food Intake

Results of the characterization of the extruded products can be found in Supplementary Table S1. Consumption of placebo vs. extruded wheat bran did differentially affect neither the background's diet fiber, carbohydrates, fat, sugar, or protein content nor total consumed calories (Supplementary Material). Macronutrient intake throughout the study period is reported in Supplementary Table S3.

### Extruded WB Increased Serum Acetate and Butyrate but Did Not Modulate Fecal SCFAs

**Figure 2:**
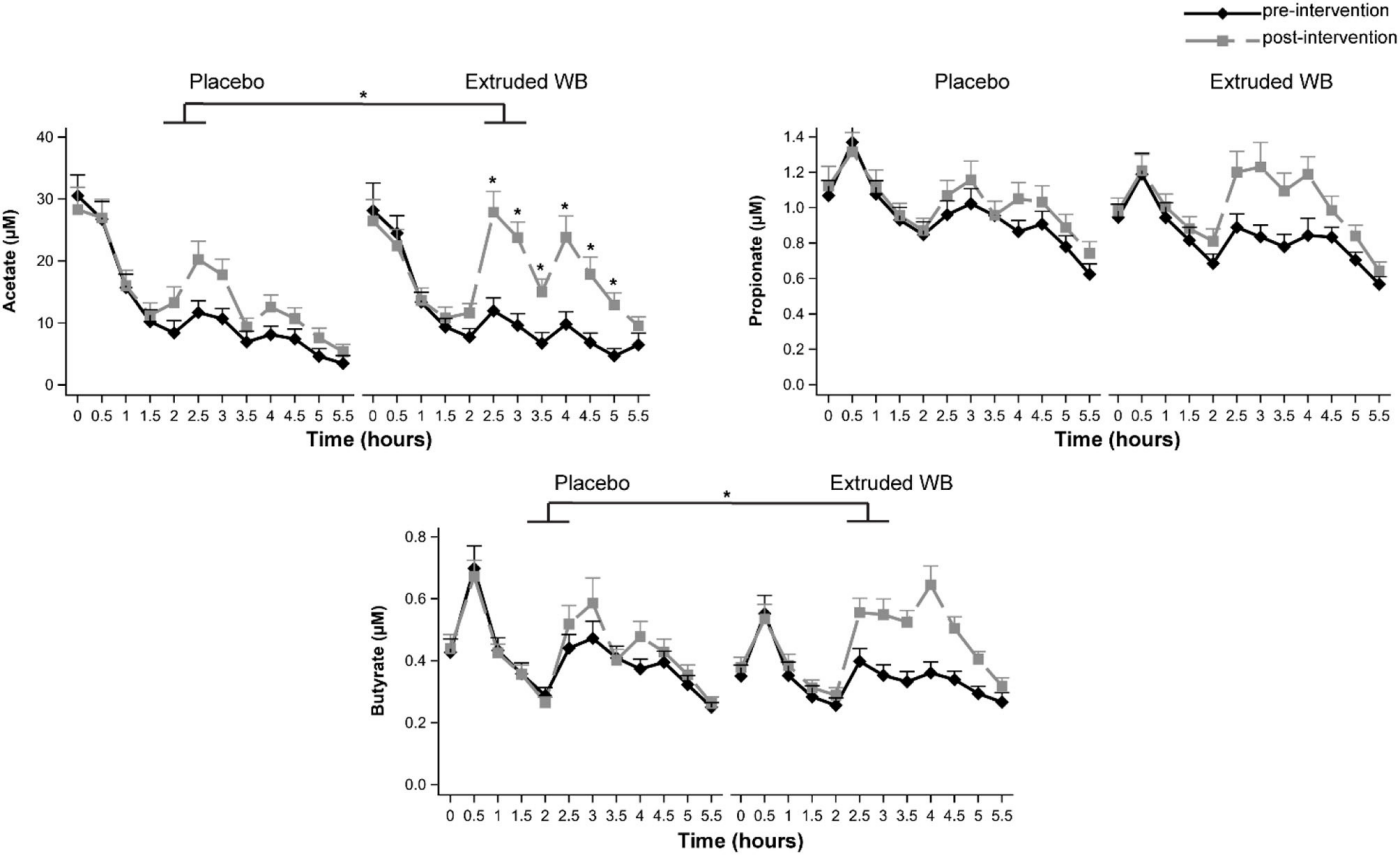
Serum short-chain fatty acid (SCFA) concentrations at pre- and post-intervention visits. Blood samples were collected throughout both study visits at 30-min intervals. Consumption of extruded wheat bran (WB) resulted in significant increases in acetate and butyrate (all *p* < 0.05), which were most pronounced from 2.5 to 5 h. Graphs show mean SCFA concentrations ± *SE*. *p < 0.05.

Consumption of extruded WB had different effects on serum SCFA concentrations relative to placebo as indicated by a significant group × visit × timepoint interaction effect for acetate (*F*_(11,680)_ = 1.96, *p* = 0.03, $\eta_{\text{p}}^{2}$ = 0.014) and a significant group × visit interaction for butyrate (*F*_(1,63)_ = 5.02, *p* = 0.029, $\eta_{\text{p}}^{2}$ = 0.003) but not for propionate (group × visit × timepoint *F*_(11,679)_= 1.54, *p* = 0.11; group × visit *F*_(1,63)_ = 0.87, *p* = 0.36). Planned contrasts per group for acetate revealed a significant increase in SCFAs from pre- to post-intervention visits starting from 2.5 to 5 h (all *p* < 0.001) following extruded WB consumption. Fasting SCFAs (time 0 h) did not differ from pre- to post-intervention in either group, indicating that chronic extruded WB consumption does not affect fasting serum SCFAs. For the placebo group, acetate increased at 2.5 h only (*p* = 0.041). Butyrate increased from pre- to post-intervention following extruded WB consumption (*p* = 0.001) but not following placebo consumption (*p* ?). The effects of fermentation of the extruded WB became apparent after at least 2 h and 30 min, reflecting the time needed to arrive in the colon (Figure 2).

Changes in fecal SCFAs were similar across the groups from pre- to post-intervention [no group × visit interaction effects for acetate (*F*_(1,62)_= 0.29, *p* = 0.59) and propionate (*F*_(1,62)_ = 0.08, *p* = 0.78) but not butyrate (*F*_(1,62)_ = 0, *p* = 0.98; Table 1)]. However, post-intervention, fecal SCFAs were lower in both groups in comparison to pre-intervention [significant main effect of visit for acetate (*F*_(1,62)_= 7.47, *p* = 0.008, $\eta_{\text{p}}^{2}$ = 0.052) and propionate (*F*_(1,62)_ = 4.67, *p* = 0.035, $\eta_{\text{p}}^{2}$ = 0.033) but not for butyrate (*F*_(1,62)_ = 2.25, *p* = 0.14)].

**Table 1:** Effects of intervention on fecal short-chain fatty acids (SCFAs), serum brain-derived neurotrophic factor (BDNF), intestinal permeability, and fecal dry weight.

		**Extruded WB**		**Extruded PL**		***P_***Interaction***_***
		**Pre-intervention**	**Post-intervention**	**Pre-intervention**	**Post-intervention**	
Fecal SCFAs (mM)		*N =* 33	*N =* 33	*N =* 31	*N =* 31	
	*Acetate*	67.87 (17.16)	61.86 (16.51)	56.21 (18.55)	48.96 (21.70)	*0.59*
	*Propionate*	17.82 (5.82)	15.64 (6.17)	13.47 (5.96)	12.06 (6.11)	*0.78*
	*Butyrate*	16.09 (6.62)	15.03 (8.17)	13.89 (9.05)	12.54 (9.39)	*0.98*
	*Total SCFAs*	101.78 (25.85)	92.53 (27.59)	83.56 (31.18)	73.56 (34.28)	*0.77*
Serum BDNF (ng/mL)		*N =* 33	*N =* 32	*N =* 33	*N =* 33	
		28.75 (7.86)	29.43 (9.15)	31.31 (8.35)	30.76 (8.07)	*0.11*
Intestinal permeability		*N =* 33	*N =* 29	*N =* 29	*N =* 30	
	*LMR*	0.08 (0.03)	0.10 (0.09)	0.08 (0.04)	0.11 (0.06)	*0.23*
		*N =* 32	*N =* 29	*N =* 30	*N =* 28	
	*FEL*	0.20 (0.18)	0.15 (0.14)	0.18 (0.13)	0.19 (0.13)	*0.12*
		*N =* 32	*N =* 33	*N =* 33	*N =* 29	
	*FEM*	13.15 (10.28)	8.86 (3.00)	12.36 (8.01)	9.27 (3.98)	*0.93*
		*N =* 34	*N =* 34	*N =* 35	*N =* 35	
Fecal dry weight (%)		26.66	26.99	28.74	34.99	*0.010*

### Extruded WB Did Not Influence Serum BDNF Concentrations

Serum BDNF levels were not affected in the current study (group × visit interaction, *F*_(1,63)_= 0.45, *p* = 0.5) (Table 1).

### Extruded WB Did Not Attenuate Responses to Acute Psychosocial Stress

**Figure 3:**
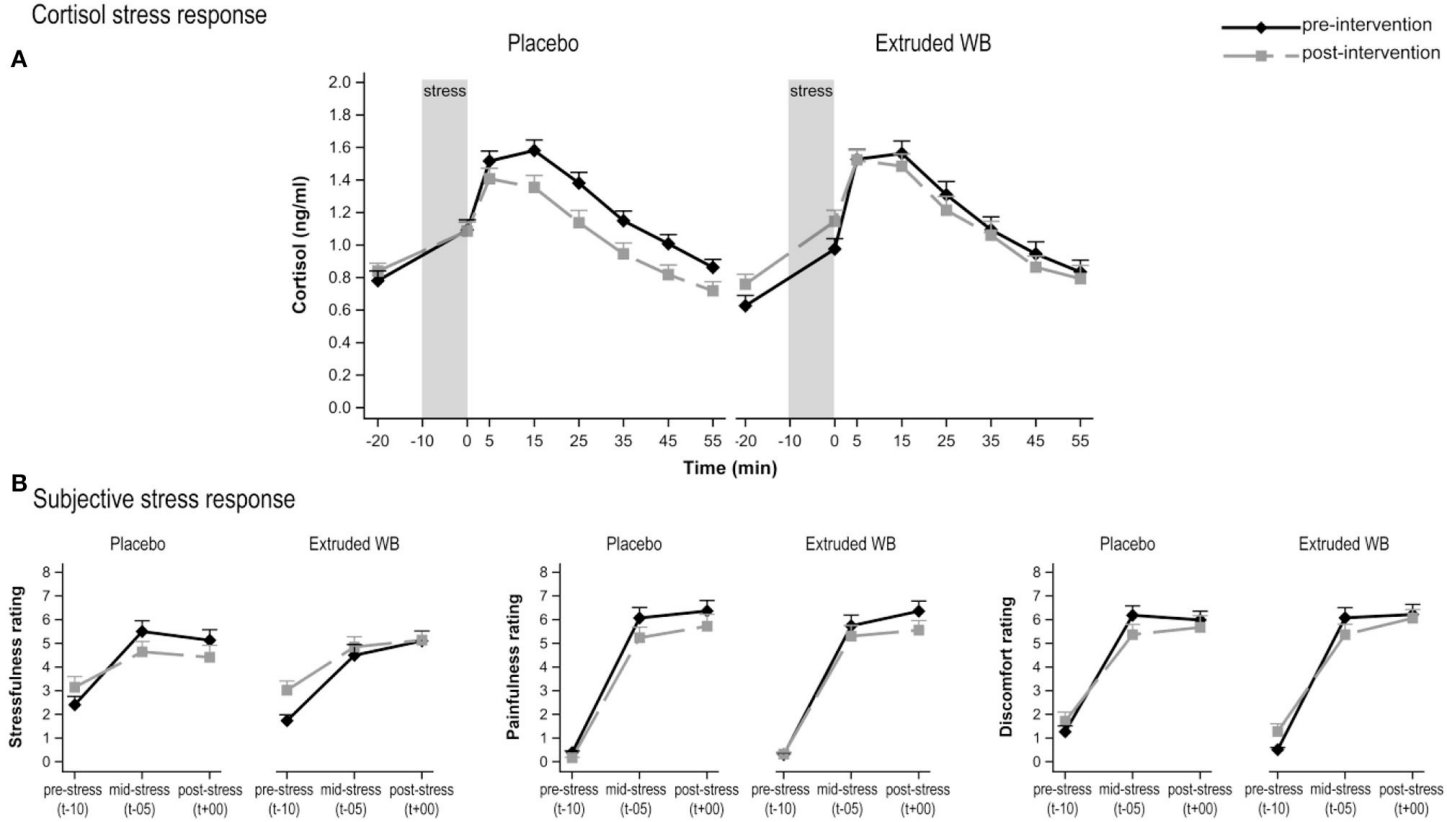
Responses to the Maastricht Acute Stress Test (MAST) at pre- and post-intervention. **(A)** Cortisol responses to acute psychosocial stress induction procedure. Saliva samples were collected from 20 min before the stress induction, up to 55 min after stress induction. Consumption of placebo vs. extruded WB did not modulate cortisol response (*p* = 0.07). **(B)** Subjective responses to acute psychosocial stress induction. Consumption of placebo vs. extruded WB did not modulate subjective ratings of stressfulness, painfulness, or discomfort in the middle and after the MAST relative to baseline values. Graphs show means of Box-Cox transformed cortisol (transformed_cortisol = [(cortisol + 1)**-025 – 1)/−0.25] concentrations and means of subjective ratings ± SE.

Consumption of extruded WB vs. placebo did not influence the cortisol response to acute stress (group × visit interaction effect (*F*_(1,64)_= 3.48, *p* = 0.07; group × visit × sample-timepoint interaction effect (*F*_(7,433)_= 1.34, *p* = 0.23); Figure 3A).

Subjective VAS responses to acute stress did not differ between the groups from pre- to post-intervention on ratings of stress (group × visit interaction effect *F*_(1,67)_= 3.17, *p* = 0.08) and pain (*F*_(1,67)_= 0.27, *p* = 0.61) but not discomfort (*F*_(1,67)_= 0.25, *p* = 0.62), neither at baseline, in the middle, nor at the end of the task (as indicated lack of group × visit × timepoint interaction effect for stress (*F*_(2,134)_= 0.51, *p* = 0.6), pain (*F*_(2,134)_= 0.78, *p* = 0.46), and discomfort (*F*_(2,134)_ = 0.11, *p* = 0.9) (Figure 3B).

### Extruded Wheat Bran Did Not Affect CAR

**Figure 4:**
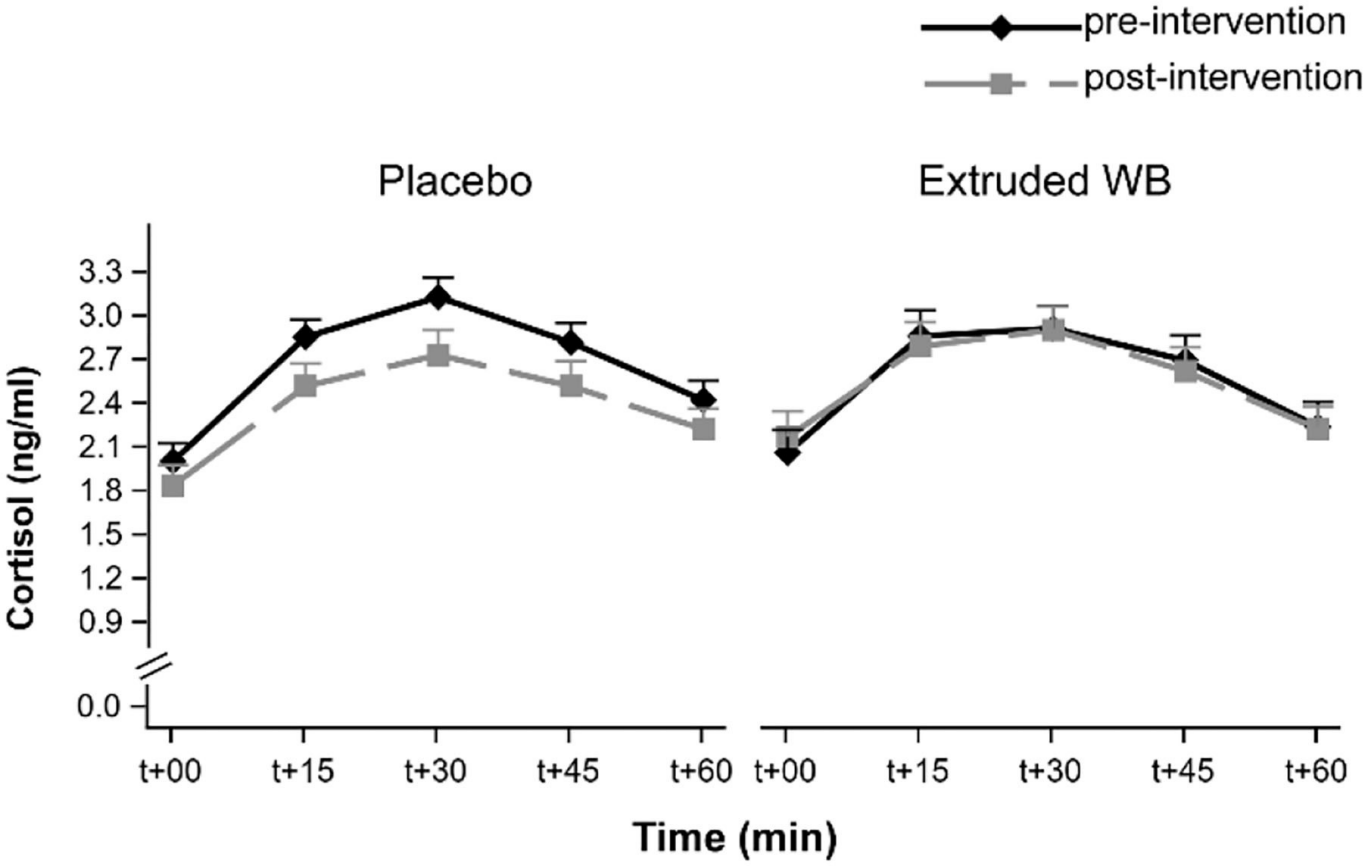
Cortisol awakening response (CAR) at pre- and post-intervention visits. CAR was not modulated by consumption of placebo nor extruded WB. T+00 represents time upon waking. Graphs show the means of transformed cortisol [transformed_cortisol = ((cortisol + 1)**0.25–1)/0.25] concentrations ± SE.

Cortisol levels rose upon waking (main effect of timepoint (*F*_(4,263)_ = 59.19, *p* < 0.001). However, the intervention did not affect CAR [group × visit interaction effect (*F*_(1,65)_ = 1.7, *p* = 0.2) and group × visit × timepoint interaction effect (*F*_(4,254)_ = 0.38, *p* = 0.82)] (Figure 4).

### Extruded Wheat Bran Did Not Modulate the Return of Fear

**Figure 5:**
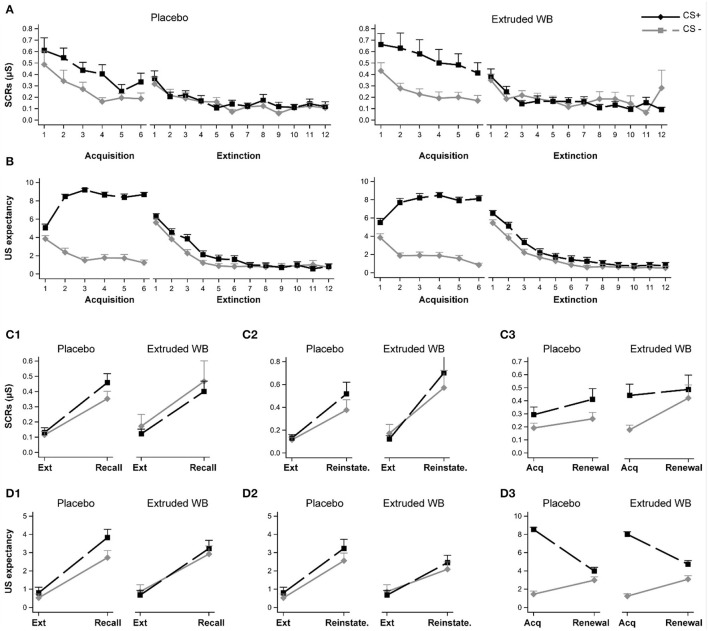
Responses to the fear conditioning and extinction task. Participants underwent fear acquisition and extinction learning pre-intervention and return of fear were tested post-intervention by examining recall of extinction memory, reinstatement of extinction memory after the unsignaled US, and fear renewal. Participants in the placebo and extruded WB group exhibited similar fear acquisition and extinction patterns as indexed by the skin conductance responses **(A)** and subjective US expectancy ratings **(B)**. Consumption of placebo or extruded WB did not modulate skin conductance responses when testing recall of fear extinction memory **(C1)**, reinstatement of fear extinction memory **(C2)**, or fear renewal when the CSs were presented in the acquisition context **(C3)**. Similarly, subjective reports of US expectancy ratings were not modulated post-intervention **(D1–D3)**. Graphs show means ± *SE*. Ext: Extinction, Reinstate.: Reinstatement, US: unconditional stimulus, SCRs: skin conductance responses.

At pre-intervention, both groups exhibited similar fear acquisition and fear extinction learning as indexed by SCRs and US expectancy ratings to the CS+ and the CS–. Furthermore, a comparison between the end of acquisition and the end of extinction indicated that both groups exhibited successful fear of extinction (Figures 5A,B; see Supplementary Material for a full analysis).

To test the effects of intervention on recall of extinction learning, the average of the last two trials of extinction learning were compared to the average of the first two trials of the recall phase 28 days later. Participants exhibited higher SCRs and US expectancy ratings to both the CS+ and the CS– during recall relative to the end of extinction learning [main effect of phase (SCRs: *F*_(1,66)_ = 76.68, *p* < 0.0001; US expectancy: *F*_(1,66)_ = 82.58, *p* < 0.0001)], but SCRs were not different between CS+ or CS– [main effect of CS (*F*_(1,67)_ = 0.58, *p* = 0.45)], whereas US expectancy ratings were higher with overall ratings for CS+ relative to CS– (main effect of CS, *F*_(1,67)_ = 4.66, *p* = 0.034). No differential CS+ vs. CS– responses were detected between the phases (CS × phase effect, SCRs: (*F*_(1,65)_ = 0.61, *p* = 0.44); US expectancy (*F*_(1,66)_ = 2.05, *p* = 0.16)]. Consumption of extruded WB or placebo did not modulate recall of extinction learning [no group × phase interaction effect, SCRs: (*F*_(1,66)_ = 0.07, *p* = 0.79); US expectancy: (*F*_(1,66)_ = 0.06, *p* = 0.8); or group × CS × phase (SCRs: *F*_(1,65)_ = 1.28, *p* = 0.26); US expectancy: (*F*_(1,66)_ = 0.83, *p* = 0.37)] (Figures 5C1,D1).

To test the effects of intervention on reinstatement of fear in the extinction context, the average of the last two trials of extinction learning were compared to the first trial following the delivery of three un-signaled USs. Participants exhibited higher SCRs and US expectancy ratings to both the CS+ and the CS– during the reinstatement trial relative to the end of extinction learning (main effect of phase (SCRs: *F*_(1,66)_ = 41.76, *p* < 0.0001; US expectancy: *F*_(1,66)_ = 50.64, *p* < 0.0001), but no differences between CS+ or CS– were detected (main effect of CS (SCRs: *F*_(1,67)_ = 1.59, *p* = 0.21; US expectancy: *F*_(1,67)_ = 2.52, *p* = 0.12) and CS × phase effect [SCRs: *F*_(1,65)_ = 1.96, *p* = 0.17; US expectancy: *F*_(1,64)_ = 0.81, *p* = 0.37]. Consumption of extruded WB or placebo did not modulate reinstatement of fear in extinction context (no group × phase (SCRs, *F*_(1,66)_ = 0.15, *p* = 0.7; US expectancy, *F*_(1,66)_ = 0.34, *p* = 0.56); nor group × CS × phase interaction effect (SCRs, *F*_(1,65)_ = 0.04, *p* = 0.84; US expectancy, *F*(_1, 64)_ = 0.13, *p* = 0.72; Figures 5C2,D2).

To test the effects of the intervention on fear renewal in the acquisition context, the average of the last two trials of acquisition were compared to the average of the first two trials of the renewal phase. SCRs to both the CS+ and the CS– did not differ during the renewal phase relative to end of acquisition (main effect of phase (*F*_(1,66)_ = 2.61, *p* = 0.11), but across both phases, SCRs were higher for CS+ relative to CS– [main effect of CS (*F*_(1,67)_ = 12.37, *p* = 0.001); CS × phase effect (*F*_(1,65)_ = 1.83, *p* = 0.18)]. For the US expectancy rating, the picture was slightly different, with a main effect of phase (*F*_(1,66)_ = 0.56, *p* = 0.003) revealing higher ratings during the end of acquisition relative to renewal (Figure 5D3). This also differed depending on the CS (*F*_(1,66)_ = 84.35, *p* < 0.0001). While CS+ and CS– differed from one another in both phases, the magnitude of this difference was larger at the end of acquisition (*t*_(66)_ = −20.92, *p* < 0.0001) relative to the renewal phase (*t*_(66)_ = −2.72, *p* = 0.008). Consumption of extruded WB or placebo did not modulate renewal of fear in the acquisition context (no group × phase interaction effect (SCRs, *F*_(1,66)_ = 0.02, *p* = 0.9; US expectancy, *F*_(1,66)_ = 3.08, *p* = 0.08); or group × CS × phase interaction effect (SCRs, *F*(_1, 65)_ = 3.20, *p* =0.08; US expectancy, *F*_(1,66)_ = 0.44, *p* = 0.51) (Figures 5C3,D3).

### Extruded WB Did Not Alter Baseline Heart Rate Variability nor Subjective Mood Ratings

The intervention did not affect HRV as indexed by RMSSD (group × visit interaction effect (*F*_(1,67)_= 0.8, *p* = 0.37). However, the expected negative association between HRV and subsequent cortisol stress response (47) was found (see Supplementary Material).

The intervention did not affect subjective ratings on the PANAS [negative affect (*F*_(1,67)_= 0.29, *p* = 0.59), positive affect (*F*_(1,67)_ = 1.26, *p* = 0.27)], PSS (*F*_(1,67)_ = 0.2, *p* = 0.65), or the DASS-21 [depression (*F*_(1,66)_ = 0.41, *p* = 0.52), anxiety (*F*_(1,66)_ = 0.74, *p* = 0.39), and stress (*F*_(1,64)_= 0.29, *p* = 0.59)]. Similarly, the intervention did not affect cognitive reactivity to sad mood as reported in the LEIDS-R Hopelessness/Suicidality subscale (*F*_(1,66)_ = 3.86, *p* = 0.054), Acceptance/Coping subscale (*F*_(1,65)_ = 0.16, *p* = 0.69), Aggression subscale (*F*_(1,67)_ = 0.77, *p* = 0.38), Perfectionism/Control subscale (*F*_(1,67)_ = 1.26, *p* = 0.27), Risk Aversion subscale (*F*_(1,67)_ = 0.27, *p* = 0.6), and Rumination subscale (*F*_(1,67)_ = 0.17, *p* = 0.68) but reflected in a composite total score (*F*_(1,67)_ = 0.07, *p* = 0.8).

### The Intervention Did Not Alter Small Intestinal Permeability but Modulated the Percentage of Fecal Dry Weight

The intervention did not influence small intestinal permeability based on LMR (*F*_(1,52)_ = 1.5, *p* = 0.23), FEL (*F*_(1,50)_ = 2.56, *p* = 0.12), or FEM (*F*_(1,57)_ = 0.02, *p* = 0.88) (Table 1).

Consumption of placebo vs. extruded WB differentially affected percentage of fecal dry weight (group × visit interaction effect (*F*_(1,67)_= 7.03, *p* = 0.01, $\eta_{\text{p}}^{2}$ = 0.049). Planned contrasts revealed a significant increase in the percentage of fecal dry weight from pre- to post-intervention visit (from 28.74 to 34.99%) in the placebo group (*t*_(67)_ = −4.12, *p* = 0.0002) but not in the extruded WB group (*t*_(67)_ = −0.34, *p* = 0.74) (from 26.66 to 26.99%) (Table 1).

The intervention also did not affect total GSRS score (*F*_(1,67)_ = 1.62, *p* = 0.21), indigestion scores (*F*_(1,67)_ = 0.78, *p* = 0.38), abdominal pain scores (*F*_(1,66)_ = 3.79, *p* = 0.06), reflux scores (*F*_(1,67)_ = 0.53, *p* = 0.47), and constipation (*F*_(1,66)_ = 0.37, *p* = 0.55). However, there was an effect on reported diarrhea (*F*_(1,67)_ = 5.52, *p* = 0.022, $\eta_{\text{p}}^{2}$ = 0.039), which tended to increase from pre- to post-intervention in the extruded WB group (*t*_(67)_ = −2.21, *p* = 0.06), but this effect did not survive correction for multiple testing comparisons.

## Discussion

This randomized, triple-blind, placebo-controlled trial in healthy men evaluated the effect of consuming a breakfast cereal consisting of extruded WB for 28 days on psychobiological outcomes. Cross-sectional and prospective cohort studies support a positive association between higher dietary fiber intake and better cognitive (5) and affective functioning (4, 48). However, previous systematic reviews of intervention studies found inconclusive evidence for their effects on mood and cognition (49, 50). Unfortunately, SCFAs are often not quantified in the systemic circulation in such dietary intervention studies, preventing an assessment of SCFAs' contribution to the psychobiological function of interest and assessing knowledge on the minimal required increase in SCFA concentrations to observe an effect of a dietary fiber intervention (6). Previously, we found that direct administration of SCFAs to the colon of healthy male participants attenuated the cortisol stress response. While extruded WB consumption in the present study significantly increased the systemic concentrations of SCFAs, we could not detect an effect on the stress response. Furthermore, no changes were observed in the CAR, return of fear processes that underlie the development of fear and anxiety symptoms, HRV, subjective mood ratings, or intestinal permeability. The placebo group exhibited increased fecal dry weight without concomitant reports of constipation.

We measured serum SCFA concentrations to determine the change in circulating acetate, propionate, and butyrate following extruded WB consumption. Postprandial acetate, butyrate, and total SCFAs, but not propionate, significantly increased from pre- to post-intervention visit in the extruded WB group relative to the placebo group. This increase was most pronounced from 2.5 to 5 h following extruded WB consumption, which coincides with likely arrival in the cecum and overlaps with the period during which the psychobiological outcomes were measured. The numerical but not significant increase in propionate concentrations can be explained by the lower proportion of propionate expected to accumulate in the colon following AX fermentation as shown previously *in vivo* (51). Previous research has shown that consumption of 60 g of an extruded bran cereal product daily for 1 year also resulted in similarly modest postprandial increases in SCFAs (52). These findings confirm that consumption of extruded WB results in limited increases in SCFA uptake in the systemic circulation.

Notwithstanding the statistically significant result, the magnitude of the increase in SCFAs was rather minimal, as evidenced by small effect sizes (partial $\eta_{\text{p}}^{2}$ = 0.003 up to 0.014). In our previous study (8), administering SCFAs directly to the colon using colon delivery capsules resulted in a markedly larger increase in circulating SCFAs, with effect sizes ranging from $\eta_{\text{p}}^{2}$ = 0.107 to 0.366. Furthermore, it resulted in a decrease in the cortisol response to the MAST, at least partially due to having attained substantial increases in serum SCFAs (8), in line with previous findings in animals using prebiotics (53–56) and SCFAs (57). Possibly, the absence of change in cortisol responses to stress in the present study is due to the relatively low fermentability of extruded WB and subsequent smaller increases of serum SCFAs. Similarly, administration of 12.5 g of a specific dietary fiber supplement, comprising more than 90% polydextrose, did not impact cortisol response to a similar acute stress task (58). Due to its complex structure, polydextrose is only partially and gradually fermented in the large intestine (59); however, serum SCFAs were not quantified in this study. To the best of our knowledge, only one other study found that administration of breakfast cereal with WB exhibited positive effects on mood and lowered self-reported stress and mental fatigue (60). However, this study lacked a placebo-comparator, limiting the confidence in its results. Taken together, it is possible that chronic (>28 days) administration of larger amounts of more highly fermentable fiber may be critical to modulating HPA-axis reactivity to stress and affective variables in general as concluded in the narrative synthesis by Desmedt and colleagues (49). Whether such a strategy would be effective by substantially increasing circulating SCFA concentrations requires systematic measurements of these metabolites in the blood in future work. Of note, the administered amount of extruded WB in the present study was already 50 g, which is high in the daily consumption range. Furthermore, the proportion of SCFAs produced from different fibers may differ and may influence psychobiological responses.

The lack of an effect of treatment on fecal SCFAs suggests complete absorption of the colonically produced SCFAs following fermentation of extruded WB as background fiber intake remained stable from pre- to post-intervention visits across both groups (see Supplementary Material). These results confirm our previous observation that administering SCFAs directly to the colon of participants did not influence fecal concentrations. This is further in line with other prebiotic studies using whole-grain wheat cereals, inulin, and polydextrose (61). Therefore, circulating SCFAs seem more informative than fecal SCFAs and seem a better proxy of colonic SCFA production, even though circulating SCFAs are subject to colonic, splanchnic, and hepatic extraction and are influenced by dietary fiber intake. Furthermore, their presence in the systemic circulation permits their interactions with other peripheral organs to influence metabolic and brain health (61). Correlations of fecal SCFAs with neuropsychiatric symptoms or psychological readouts in healthy populations and patients with mental disorders (6) are likely to be irrelevant and should be discouraged.

Extruded WB did not modulate the CAR, BDNF levels, or fear responses in human. Previous study showed that administration of 5 g/day of galactooligosaccharides (GOS) for 3 weeks significantly decreased the CAR in healthy male participants (62) and that fasting plasma BDNF levels were increased by 27%, 10.5 h following ingestion of whole-grain rye bread in healthy men and women (24). Findings in rodents point to the role of the gut microbiota (63, 64) and butyrate production (65) in modulating fear responses by modulating amygdala-dependent brain functions (66) and epigenetic modulation of memory processes underpinning fear through enhanced histone acetylation (65), respectively. Our previous study using colon delivery capsules revealed that SCFAs are likely not responsible for the putative effects of dietary fiber or prebiotic fermentation on the CAR, BDNF, or fear responses (8), at least in the administered doses and the specific acetate:propionate:butyrate ratio used. Extruded WB consumption may have resulted in additional metabolic effects beyond SCFA production, such as (a) acting as a prebiotic and resulting in changes in microbiota composition and (b) prompting the release of phenolic compounds, such as ferulic acid, into the bloodstream (67, 68). In addition, *in vitro* results of the extruded WB samples showed degradation of phytate, which increased the solubility and absorption of minerals present in WB (21, 22), which can be relevant for the regulation of the HPA-axis, anxiety, and BDNF levels (69–71). Presuming that such changes occurred *in vivo* in our participants, the lack of effects on CAR, BDNF, and fear responses suggests that more research is needed to understand the mechanisms and the dose and proportion of different SCFAs needed to modulate these readouts. On a different note, BDNF and fear responses are tied to butyrate-induced HDAC inhibition (72). Notwithstanding that the effects of acetate and propionate have been much less extensively studied relative to butyrate, it is possible that a substantial increase of butyrate alone, perhaps without the interference of the effects of other SCFAs, is needed to modify these parameters in human.

Extruded WB consumption did not affect other outcomes, such as subjective mood ratings, HRV, or intestinal permeability. While this is in contrast to previous RCTs using prebiotics (49), where effects were predominantly demonstrated on self-report mood measures, our cohort exhibited normal scores at pre-intervention on subjective reports of mood and emotional states, and the intervention did not improve or worsen them. This is also in line with our previous study using colonic SCFA administration, which conducted the intervention on a sample of the same population (8). It is noteworthy that the previously-observed effects on subjective mood scores were evident only with prebiotic interventions for either (a) cohorts with pre-existing ailments (e.g., IBS, diabetes) or mental health disorders (e.g., depression) or for (b) healthy participants who consumed prebiotics for a prolonged period of time (e.g., minimum three weeks) (49, 73). It is therefore tempting to speculate that longer-term highly fermentable-fiber consumption (as a proxy for continuous SCFA production) may be needed to influence subjective mood.

High HRV, as a measure of the vagal tone, is considered a marker of successful emotion regulation and adaptability to stress (74). Low resting and pre-stress HRV have both been associated with higher stress-induced cortisol levels and delayed recovery of cardiovascular, endocrine, and immune responses to acute stressor (47, 75). Therefore, we measured resting HRV to test whether it is also modulated by the intervention and would aid in understanding the hypothesized differences in the stress response. The intervention, however, did not modulate resting HRV. Yet, we were able to replicate previously reported associations at pre-intervention, namely that higher resting HRV is associated with lower cortisol stress response and recovery (see Supplementary Figure S1).

Consumption of dietary fiber may be one of the few dietary interventions with the potential to reinforce a healthy intestinal barrier (76), for instance by (a) preventing glycan-consuming microbiota from degrading the mucus layer's integrity or (b) via the production of SCFAs that regulate mucosal immune barrier function (77). However, our sample exhibited normal levels for indices of intestinal permeability (LMR, FEL, FEM) that were not altered following consumption of extruded WB. This suggests that a protective effect on intestinal permeability may only be observed in subjects with increased intestinal permeability, such as after a stress challenge (45).

In relation to the local effects of the intervention products, consumption of the placebo product resulted in an increase in the percentage of fecal dry weight from pre- to post-intervention visits (from 28.74 to 34.99%). This is in line with a previous study showing that consumption of microcrystalline cellulose increases fecal dry matter from about 28 to 35% in dogs (78). However, neither of the interventions' influences reported gastrointestinal symptoms, suggesting that they were well tolerated.

The strengths of the current study include intensive profiling of SCFAs in the systemic circulation and limiting the effects of various confounding factors highlighted in a previous meta-analysis (50), including controlling for gender, dietary (particularly fiber) intake, and weight. In addition, we have utilized experimental paradigms to assess psychobiological functioning beyond the sole reliance on self-reported measures (49), as the latter is prone to be driven by socially-desirable responses. However, the current study did not analyze gut microbiota composition, which limits assessing the extent to which the intervention modulated the abundance of SCFA-producing bacteria. Variability in changes or even a potential lack of changes in gut microbiota composition might have underlined the null findings on psychobiological functioning. Finally, floor and ceiling effects in potentially already “healthy” gut microbiota may limit the impact on psychobiological outcomes, as proposed previously (49).”

In conclusion, our findings indicate that extruded WB increases serum SCFA levels; however, this increase was not sufficiently large in magnitude to elicit measurable effects on psychobiological markers in healthy male subjects. Establishing whether dietary fiber interventions exert their effects via SCFAs requires additional work to uncover the minimal necessary increase in SCFAs. Rather than attempting to induce health benefits with a single fiber-rich food, combinations of different types of fermentable fibers, particularly highly fermentable ones, might be needed to substantially increase SCFA production and systemic concentrations. The minimally required increase in SCFAs and the proportion of individual SCFA required to induce an effect may vary for different health benefits related to carbohydrate fermentation or in different populations (with and without psychopathologies) and should be investigated for each endpoint and population of interest.

## Data Availability Statement

The raw data supporting the conclusions of this article will be made available by the authors, without undue reservation.

## Ethics Statement

The studies involving human participants were reviewed and approved by Medical Ethics Committee of UZ Leuven/KU Leuven. The patients/participants provided their written informed consent to participate in this study.

## Author Contributions

BD: conceptualization, methodology, software, formal analysis, investigation, data curation, writing-original draft, writing-review & editing, visualization, and project administration. DL, PK, and FZ: investigation and writing-review & editing. CR: conceptualization, investigation, and writing-review and editing. CL and LL: writing-review and editing. GB: conceptualization, resources, and writing-review and editing. CC: conceptualization, resources, writing-review and editing, supervision, and funding acquisition. BV: conceptualization, methodology, writing-review and editing, and supervision. LO and KV: conceptualization, methodology, resources, writing-review and editing, supervision, and funding acquisition. All authors contributed to the article and approved the submitted version.

## Funding

This study was supported by an infrastructure grant from Herculesstichting, Belgium (AKUL/13/07). The PhDs of Boushra Dalile and Chiara Roye are financially supported by an unrestricted grant from the Société des produits Nestlé S.A. This study received funding from Societé des Produits Nestlé S.A. The funder had the following involvement with the study: a) manufactured test samples used to conduct the clinical study and b) provided funding to conduct the research project at KU Leuven.

## Conflict of Interest

CL and LL are currently employed by Societé des Produits Nestlé S.A. GB was employed by Societé des Produits Nestlé S.A. at the time when the work was conducted. The remaining authors declare that the research was conducted in the absence of any commercial or financial relationships that could be construed as a potential conflict of interest.

## Publisher's Note

All claims expressed in this article are solely those of the authors and do not necessarily represent those of their affiliated organizations, or those of the publisher, the editors and the reviewers. Any product that may be evaluated in this article, or claim that may be made by its manufacturer, is not guaranteed or endorsed by the publisher.
