# Supplementary material for: Extruded Wheat Bran Consumption Increases Serum Short-Chain Fatty Acids but Does Not Modulate Psychobiological Functions in Healthy Men: A Randomized, Placebo-Controlled Trial
Source: Front Nutr. 2022 May 26;9:896154. doi: 10.3389/fnut.2022.896154 (PMC9178292; doi:10.3389/fnut.2022.896154)
Supplement: Supplementary file 1 [file Data_Sheet_1.docx]

**SUPPLEMENTAL INFORMATION**

**MATERIALS AND METHODS**

**Randomization and allocation**

The online randomization tool (<http://www.randomization.com/>) was used to randomize participants into three groups. Seventy-two subjects were randomized into blocks of 10, 10, 10, 10, 10, 10, 2, 10. The randomization of placebo and bran products and their blinded assignment to each volunteer was performed by Greet Vandermeulen, a collaborator in the Laboratory of Digestion and Absorption, who is not involved in the study. Bags were sequentially numbered by Ms Vandermeulen and provided to Boushra Dalile, who enrolled the participants and tested them. The bags were not transparent and were simply handed by Boushra Dalile to each participant. Since the study employs a parallel group design, it was impossible for participants in the study to discern which treatment group they belong to. Group names remained encrypted, and thus all parties involved in the study remained blinded, until the entire data collection, pre-processing of samples and psychophysiological data, and statistical analysis was completed (i.e., triple-blind).

**Power calculation**

The primary outcome of the study is the participants’ cortisol stress response. Therefore, a power calculation was done based on data obtained from our previous work^1^ from the stress-induction protocol (MAST^2^). This was done using the General Linear Multivariate Model Power & Sample Size software (GLIMMPSE, <http://glimmpse.samplesizeshop.org/#/>), which relied on means and standard deviations of the cortisol stress responders^3^ in the low dose SCFA group^1^, MAST timepoints, and study visits). A total sample size of 70 participants (n=35 per treatment arm) yielded sufficient power (80%) for the current study to test the group x visit interaction effect.

**Composition of standardized meals during study visits**

For breakfast during the first study visit, participants consumed 60 grams of rice crips and 250 mL of lactose-free milk. The breakfast during the second study visit comprised the assigned intervention product. For lunch during both study visits, participants consumed slices of white bread with a choice between slices of cheese, ham, chicken, turkey, or salami, or a combination thereof.

**Fear learning and extinction task**

The Fear Conditioning and Extinction Paradigm^4^ was programmed in Affect 5.0 software and used in this study to test for the return of fear at post-intervention. Participants were presented with two neutral stimuli, one of which is repetitively paired with an aversive outcome [unconditional stimulus (mild electrical stimulation); US], while the other is never paired with a US. The mild electrical stimulation was delivered with a duration of 2 ms through two electrodes (8mm, with an inter-electrode distance of 30 mm, Digitimer, Hertfordshire, United Kingdom) to the wrist of the dominant hand by a Digitimer DS7A constant-current stimulator (Hertfordshire, United Kingdom). The intensity level of the stimulus was determined by the participants themselves to be “highly uncomfortable but not painful”.

Participants learn that one stimulus such as a blue lamp light [referred to as a positive conditional stimulus (CS+)] predicts the US and comes to elicit greater fear responses such as increases in heart rate and sweat excretion, whereas another stimulus which does not predict the US, such as a yellow lamp light, represents a safe stimulus [referred to as a negative conditional stimulus (CS-)]. To present each stimulus for each trial, an unlit lamp was shown for 3 s before being switched on to one of the three lights (blue, red, or yellow) for 6 s in one of two different background contexts [a picture with a desk and a computer (context A), and a bookshelf (context B)]. At pre-intervention visit, participants completed habituation, acquisition, and extinction learning phases. At post-intervention visit, participants completed recall, reinstatement, and renewal phases. During the habituation phase, the CS+ and CS- were each presented two times in context A and context B without the US, in order to familiarize the participants to the visual stimuli. During the acquisition phase, the CS+ and the CS- were each presented six times in the conditioning context (e.g., context A). The electrical stimulation followed the CS+ 83% of the time and was delivered at the end of the CS presentation and co-terminated with it. The CS- was never followed by the shock US. In this way, after repetitive exposure during the acquisition phase, participants acquire fear to the CS+ but not to the CS-. After the acquisition phase and during the extinction pahse, fear was extinguished by presenting 12 non-reinforced CS+ and 12 CS- trials in the extinction context (e.g., context B).

Following extinction learning at pre-intervention visit, participants were told to remember what they have learned during that task as it will continue at post-intervention visit. When participants returned at post-intervention visit, they were asked if they remember the CSs and the contexts, and in which context did they receive a shock to the CS+. The task proceeded with an extinction recall phase, where the CS+ and the CS- where presented four times each in the extinction context (context B) to test recall of extinction learning after a lapse of time, without US presentation. This was followed by a 5-minute break, after which 3 unsignalled shocks were delivered with a random interval of 12 to 16 s in between. This was followed by 1 minute pause, after which the reinstatement phase continued with 8 CS+ and 8 CS- trials in context B, without US presentation, to test whether the presentation of the unsignalled shocks (US) reinstates the original fear memory (acquired in context A) within context B. The task then proceeded to renewal phase, in which the CS+ and the CS- where presented four times each in the conditioning context (context A) without US presentation. The inter-trial interval (ITI) was randomly set per trial and ranged from 12 to 16 s.

Skin conductance was measured by attaching two pre-gelled 8-mm Ag/AgCl electrodermal activity disposable electrodes (Biopac Systems, Goleta, CA, United States) to the hypothenar eminence of the non-dominant hand using a Coulbourn isolated skin conductance coupler (LabLinc v71-23, Coulbourn Instruments). The data was sampled at 1000Hz. The raw analog signal was digitized by a 16-bit AD converter (National Instruments, NI-6221). Offline data extraction was completed with MATLAB. SCRs were calculated by subtracting the mean of the 2 ms SCL (baseline) prior to CS presentation from the maximum SCL obtained during CS presentation (6s).

US expectancy ratings were collected on a trial-by-trial basis. One second after each CS onset, a rating scale appeared on the screen requiring the participants to evaluate the likelihood of receiving an electric stimulation. Participants had to answer as quickly as possible on a scale from 0 to 10 (where 0 = no shock, 5 = maybe, 10 = definitely a shock). The scale remained for the total CS duration.

Data acquired during the acquisition and extinction phases were analyzed on a trial-by-trial basis. Furthermore, to test whether participants extinguished their fear, the last two trials of the acquisition phase were averaged and compared with the average of the last two trials of the extinction phase. To test for the effect of the intervention on recall of extinction memory, the last two trials of the extinction learning phase at pre-intervention were averaged and compared with the average of the first two trials of the recall phase at post-intervention. To test for reinstatement of the original fear memory in the extinction context after delivery of three unsignalled USs, the last two trials of the extinction phase at pre-intervention were averaged and compared with the first trial of the reinstatement phase as done previously^5^. Finally, to test the effect of intervention on return of fear in the acquisition context (i.e. fear renewal in context A), the average of the last two acquisition trials was compared with the average of the first two renewal trials. This approach was applied to test the effects of the intervention on US expectancy and SCRs.

**Questionnaires**

The positive and negative affect schedule (PANAS), perceived stress scale (PSS), depression, anxiety, and stress scales (DASS-21), and the gastrointestinal symptom rating scale (GSRS) have been extensively described in Supplemental Information elsewhere^1^. In the current study, the recall period was ‘over the past month’.

*Leiden Index of Depression Sensitivity-Revised (LEIDS-R)*

The LEIDS-R^6^ is a self-report measure consisting of 34 items that asses cognitive reactivity to sad mood. The LEIDS and its revised version, LEIDS-R, reliably distinguish between healthy and depression-vulnerable subjects. It is emphasized that the statements apply to situations when “it is certainly not a good day, but you don’t feel truly down or depressed”. In turn, the respondents are requested to indicate on a 5-point Likert-scale the extent to which each statement applied to them, where 0 represents ‘not at all’ and 7 represents ‘very strongly’.

**RESULTS**

**Characterization of the intervention products**

| **Table S1.** System parameters measured during extrusion-cooking and some physicochemical characteristic of extruded wheat bran and the extruded placebo sample (50% wheat flour and 50% MCC). Native wheat bran and control wheat bran (same particle size as extruded wheat bran) were also included. | | | | |
| --- | --- | --- | --- | --- |
|  | Extruded wheat bran | Extruded wheat flour - MCC | Native wheat bran | Milled and heat-treated wheat bran |
| *SME (kJ/kg)* | 522 | 431 | / | / |
| *Pressure (Bar)* | 65 | 55 | / | / |
| *Product T (°C)* | 149 | 127 | / | / |
| *SWBC (g/g dm)* | 1.21 (±0.03) | ND | 0.88 (±0.02) | 0.88 (±0.01) |
| *TWBC (g/g dm)* | 3.14 (±0.25) | 2.32 (±0.03) | 5.60 (±0.22) | 3.64 (±0.08) |
| *Swelling capacity (mL/g)* | 4.47 (±0.21) | 4.43 (±0.19) | 2.33 (±0.77) | 2.29 (±0.10) |
| *Extractability (% dm)* | 20.4 (±0.5) | 18.4 (±0.1) | 14.9 (±0.5) | 16.9 (±0.4) |
| Note: Abbreviations: SME, specific mechanical energy; T, temperature; SWBC: strong water-binding capacity; TWBC, total water-binding capacity; MCC, microcrystalline cellulose; ND, Not determined due to clotting of the filter. | | | | |

**Fear acquisition and extinction learning at pre-intervention visit**

At pre-intervention, both groups displayed fear acquisition, as evidenced by higher SCR to the CS+ relative to the CS- across the fear acquisition phase (main effect of CS, *F*(1,66) = 22.89, *p* < 0.0001). SCRs were attenuating throughout the acquisition phase as indicated by a main effect of trial (*F*(5,335) = 8.79, *p* < 0.0001), however this did not differ between the groups (group × trial, (*F*(5,335) = 0.61, *p* = 0.70). Differential (CS+ vs CS-) SCRs during the acquisition phase were not significantly different between the groups (group × CS (*F*(1,66) = 1.39, *p* = 0.24; group × CS × trial (*F*(5,330) = 0.78, *p* = 0.56)). A similar pattern appeared on the subjective level, with both groups reporting higher US expectancy ratings in response to the CS+ relative to the CS-, but this differed throughout the acquisition phase (CS × trial interaction (*F*(5,318) = 25.02, *p* < 0.0001). Specifically, participants reported increasing US expectancy ratings to the CS+ and decreasing US expectancy ratings to the CS- over time during the acquisition phase. These differential US expectancy ratings during the acquisition phase were not significantly different between the groups (group × CS (*F*(1,67) = 0.67, *p* = 0.42; group × CS × trial (*F*(5,318) = 0.78, *p* = 0.56)). Together, this indicates that both groups exhibited similar differential SCR responses between the CS+ and the CS- throughout the acquisition phase.

During the extinction phase, both CS+ and CS- elicited similar SCRs (main effect of CS (*F*(1,66) = 0.98, *p* = 0.32). However, as expected, SCRs decreased throughout the extinction phase as indicated by a significant main effect of trial (*F*(11,737) = 10.77, *p* <0.0001). This effect was similar for both CS+ and CS- (CS × trial (*F*(11,726) = 1.07, *p* = 0.38), and for both groups (group × CS (*F*(1,66) = 0.00, *p* = 0.95); group × trial (*F*(11,737) = 0.98, *p* = 0.47); group × CS × trial (*F*(11,726) = 1.20, *p* = 0.28)). At the subjective level, participants reported decreasing US expectancy ratings over time, but reported higher ratings for CS+ relative to the CS- (CS × trial (*F*(11,706) = 2.60, *p* = 0.003). Differential US expectancy ratings during the extinction phase were not significantly different between the groups (group × CS (*F*(1,67) = 0.03, *p* = 0.86; group × CS × trial (*F*(11,706) = 1.16, *p* = 0.31). Together, these data indicate that both groups exhibited similar responses to the CS+ and the CS- throughout the extinction learning phase.

To verify the success of fear extinction procedure, the average of the last two trials during the acquisition were compared with the average of the last two trials of extinction learning phase. There was a significant decrease in SCRs and US expectancy ratings from the end of acquisition to the end of extinction (main effect of phase: SCRs, *F*(1,67) = 26.57, *p* < 0.0001; US expectancy, *F*(1,67) = 191.34, *p* < 0.0001), which was similar across both groups (no group × phase interaction effect for SCRs (*F*(1,67) = 1.09, *p* = 0.30, nor for US expectancy ratings (*F*(1,67) = 0.08, *p* = 0.78). Furthermore, a CS × phase interaction effect for SCRs (*F*(1,66) = 7.24, *p* = 0.009) and for US expectancy ratings (*F*(1,67) = 97.49, p <0.0001) indicated higher SCRs and US expectancy ratings in response to the CS+ relative to the CS- during the end of acquisition phase (SCRs: *t*(66) = -3.35, *p* = 0.003; US expectancy: *t*(67)= -20.66, *p* < 0.0001) but not end of extinction learning (SCRs: *t*(66) = -0.11, *p* = 0.91; US expectancy: *t*(67) = -0.27, *p* = 0.79). Nevertheless, this was not different between the groups (SCRs: *F*(1,66) = 1.70, *p* = 0.20; US expectancy: *F*(1,67) = 0.02, *p* = 0.89). This indicates that participants across both groups successfully acquired fear to the CS+ relative to the CS- and successfully extinguished the fear responses by the end of the extinction training phase *(Fig 5a, b)*.

**Association between HRV and cortisol stress response**

To test the hypothesized association between HRV and cortisol response to stress, data from the pre-intervention visit were analyzed by pooling participants from both groups together (n= 68). Data from post-intervention visit were not analyzed to avoid the influence of the intervention and habituation/expectancy in relation to the procedure. HRV and cortisol levels were first transformed using Box-Cox Transformation to achieve normal distribution. A linear mixed model was run with cortisol as the dependent variable and standardized HRV and time of saliva sampling as factors, as well as their interaction. There was a significant standardized HRV × time interaction effect (*F*(7,453) = 2.69, *p* = 0.0096, η_p_^2^ = 0.034), indicating that the lower the resting HRV, the higher the cortisol stress response and recovery was (Fig S2).

**
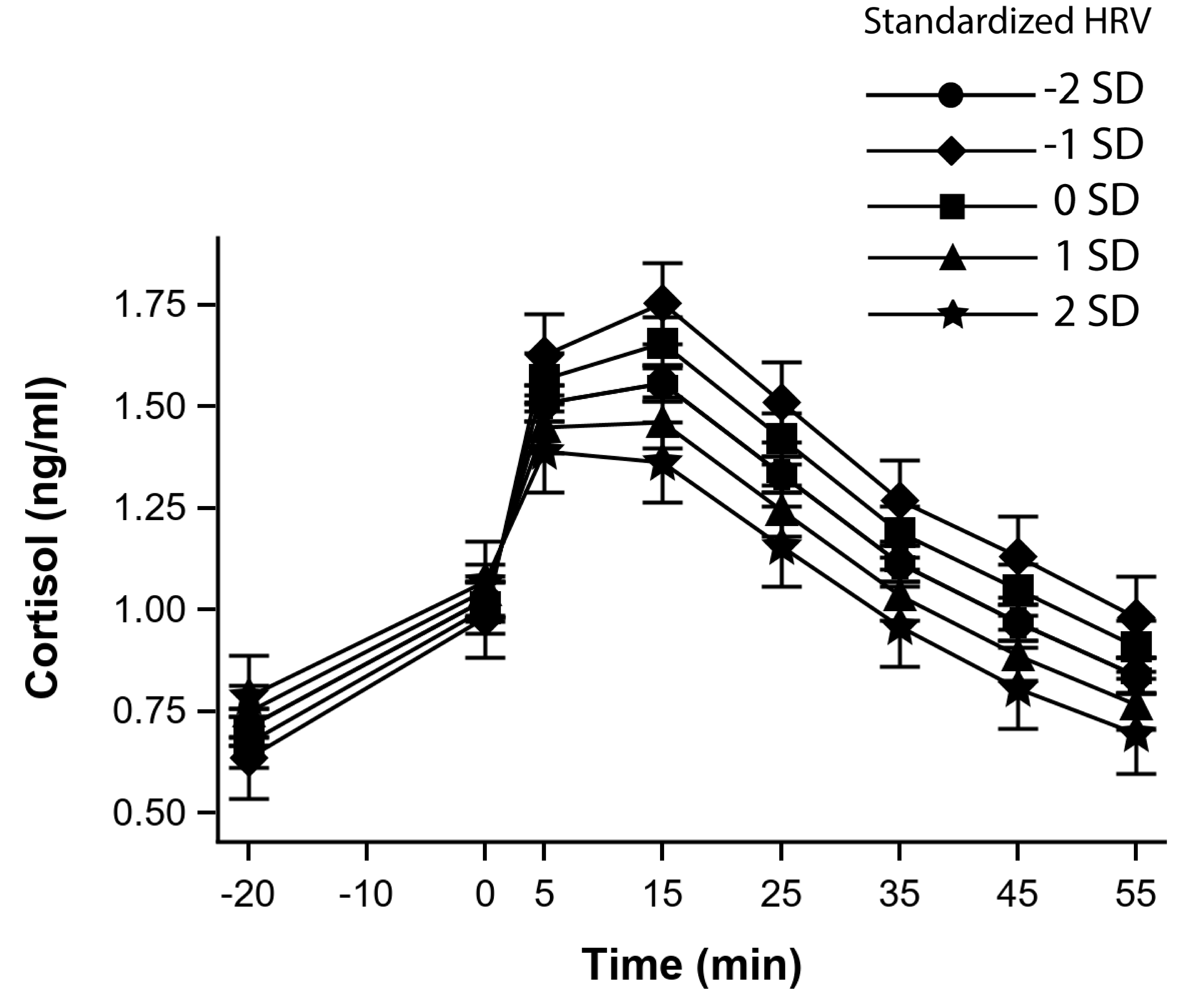
**

**Fig S1.** Cortisol levels in response to the MAST covaried significantly with resting HRV. Lower resting HRV (e.g. HRV at -2 and -1 standard deviations (SD) below the mean) were associated with higher cortisol levels during peak response and throughout the recovery period. The graph represents mean cortisol estimates ± *SE*.

**Questionnaires**

| **Table S2.** Effects of intervention on subjective mood and gastrointestinal symptom ratings | | | | | | |  |  |
| --- | --- | --- | --- | --- | --- | --- | --- | --- |
|  |  | **Placebo** | |  | **Extruded WB** | |  | *P_Interaction_* |
|  |  | Pre-intervention | Post-intervention |  | Pre-intervention | Post-intervention |  |  |
|  |  | N = 35 | N = 35 |  | N = 34 | N = 34 |  |  |
| DASS |  |  |  |  |  |  |  |  |
|  | *Depression* | 9.11(2.64) | 9.03(2.88) |  | 8.29(1.66) | 8.15(2.05) |  | *0.52* |
|  | *Anxiety* | 8.40(1.48) | 8.31(1.32) |  | 7.88(1.30) | 7.88(1.93) |  | *0.39* |
|  | *Stress* | 10.34(2.58) | 10.71(3.71) |  | 9.44(2.09) | 9.76(2.63) |  | *0.59* |
| PSS |  | 12.06(5.69) | 12.57(6.49) |  | 10.74(5.28) | 11.62(5.68) |  | *0.65* |
| LEIDS-R |  |  |  |  |  |  |  |  |
|  | *Hopelessness/suicidality* | 7.37(2.73) | 7.49(3.90) |  | 6.59(2.28) | 6.56(1.52) |  | 0.05 |
|  | *Acceptance/Coping* | 6.94(2.54) | 7.23(2.20) |  | 7.06(2.40) | 7.35(2.52) |  | 0.69 |
|  | *Aggression* | 10.26(3.43) | 11.03(4.20) |  | 9.35(2.40) | 9.47(2.85) |  | 0.38 |
|  | *Perfectionism/Control* | 12.06(2.99) | 12.11(3.32) |  | 11.56(3.92) | 12.29(3.74) |  | 0.27 |
|  | *Risk Aversion* | 13.74(3.60) | 13.74(3.99) |  | 12.62(3.63) | 12.15(3.25) |  | 0.60 |
|  | *Rumination* | 15.57(4.53) | 15.17(5.12) |  | 13.56(4.01) | 12.85(4.45) |  | 0.68 |
|  | *Total score* | 65.94(13.68) | 66.77(17.07) |  | 60.74(12.82) | 60.68(14.48) |  | 0.80 |
| PANAS |  |  |  |  |  |  |  |  |
|  | *PA* | 35.66(5.40) | 33.89(6.39) |  | 37.18(5.72) | 34.38(5.84) |  | *0.27* |
|  | *NA* | 18.51(5.22) | 17.54(5.55) |  | 17.21(5.13) | 16.59(4.53) |  | *0.59* |
| GSRS |  |  |  |  |  |  |  |  |
|  | *Abdominal pain* | 1.84(0.82) | 1.60(0.73) |  | 1.57(0.61) | 1.55(0.57) |  | *0.06* |
|  | *Reflux syndrome* | 1.21(0.39) | 1.16(0.36) |  | 1.28(0.55) | 1.18(0.32) |  | *0.47* |
|  | *Diarrhea* | 1.67(0.67) | 1.67(0.87) |  | 1.51(0.69) | 1.71(0.72) |  | ***0.022*** |
|  | *Indigestion syndrome* | 2.20(0.89) | 1.97(0.86) |  | 2.06(0.80) | 1.99(0.83) |  | *0.38* |
|  | *Constipation* | 1.50(0.53) | 1.55(0.76) |  | 1.47(0.70) | 1.37(0.63) |  | *0.55* |
|  | *Total score* | 1.75(0.52) | 1.64(0.56) |  | 1.63(0.49) | 1.61(0.49) |  | *0.21* |
| Note: Values represent means and standard deviations. DASS, depression, anxiety, and stress scales; PSS, perceived stress scale; LEIDS-R, Leiden index of depression sensitivity-revised; PANAS, positive and negative affect schedule; PA, positive affect, NA, negative affect; GSRS, gastrointestinal symptom rating scale. | | | | | | | | |

**Food diary**

Participants were instructed to note everything they eat and drink for three days during three time periods (1) at recruitment, (2) three days before pre-intervention visit, and (3) three days before post-intervention visit. Participants logged their dietary intake on [www.myfitnesspal.com](http://www.myfitnesspal.com), and these food diaries were used to calculate the participants’ macronutrient and caloric intake (Table S3). The intake of each macronutrient was averaged across the three days, and this value was entered in a linear mixed model with “group” (placebo vs. bran arms) and “period” (baseline, pre-intervention, and post-intervention) as main effects and their interaction as the effect of interest.

Consumption of placebo versus extruded wheat bran did not differentially affect the background’s diet fiber, carbohydrates, fat, sugar, or protein content, nor total consumed calories as indicated by a non-signature group x time period interaction effects (Fiber, *F*(2,129) = 0.99, *p* = 0.37; Carbohydrates, *F*(2,129) = 0.85, *p* = 0.43; Fat, *F*(2,129) = 0.28, *p* = 0.75; Sugar, *F*(2,129) = 0.19, *p* = 0.83; Protein, *F*(2,129) = 0.05, *p* = 0.95; Calories, *F*(2,129) = 0.13, *p* = 0.88).

| **Table S3.** Macronutrient intake across treatment arms at pre- and post-intervention. | | | | | | | | |
| --- | --- | --- | --- | --- | --- | --- | --- | --- |
|  | **Placebo** | | |  | **Extruded WB** | | | |
|  | Baseline period | Pre-intervention | Post-intervention |  | Baseline period | Pre-intervention | Post-intervention |  |
|  | N=33 | N=35 | N=34 |  | N=33 | N=33 | N=33 |  |
| *Fiber* | 17.77(10.09) | 13.42(6.14) | 14.12(7.21) |  | 17.15(8.56) | 14.71(8.21) | 12.31(5.35) |  |
| *Carbohydrates* | 244.14(90.03) | 239.64(81.58) | 204.50(64.62) |  | 264.32(79.43) | 235.90(78.79) | 211.02(66.74) |  |
| *Fat* | 75.30(26.74) | 69.49(23.20) | 74.16(32.10) |  | 78.40(31.00) | 70.80(23.87) | 77.36(28.17) |  |
| *Sugar* | 58.75(33.84) | 59.00(29.11) | 51.92(32.22) |  | 51.94(21.47) | 54.55(32.92) | 48.31(22.21) |  |
| *Protein* | 93.43(30.08) | 84.60(28.80) | 84.95(34.52) |  | 97.75(36.33) | 85.16(25.80) | 87.16(41.48) |  |
| *Calories* | 2009.23(454.01) | 1900.39(336.88) | 1861.85(552.99) |  | 2078.38(558.21) | 1960.46(457.53) | 1862.72(474.71) |  |
| Note: The macronutrient values represent the background diet, excluding intervention product-derived macronutrients.  Data represent means and standard deviations. | | | | | | | | |

**References**

1 Dalile, B., Vervliet, B., Bergonzelli, G., Verbeke, K. & Van Oudenhove, L. Colon-delivered short-chain fatty acids attenuate the cortisol response to psychosocial stress in healthy men: a randomized, placebo-controlled trial. *Neuropsychopharmacology : official publication of the American College of Neuropsychopharmacology* **45**, 2257-2266 (2020).

2 Smeets, T. *et al.* Introducing the Maastricht Acute Stress Test (MAST): a quick and non-invasive approach to elicit robust autonomic and glucocorticoid stress responses. *Psychoneuroendocrinology* **37**, 1998-2008 (2012).

3 Miller, R., Plessow, F., Kirschbaum, C. & Stalder, T. Classification criteria for distinguishing cortisol responders from nonresponders to psychosocial stress: evaluation of salivary cortisol pulse detection in panel designs. *Psychosom. Med.* **75**, 832-840 (2013).

4 Zeidan, M. A. *et al.* Test-retest reliability during fear acquisition and fear extinction in humans. *CNS. Neurosci. Ther.* **18**, 313-317 (2012).

5 Kindt, M., Soeter, M. & Vervliet, B. Beyond extinction: erasing human fear responses and preventing the return of fear. *Nat. Neurosci.* **12**, 256-258 (2009).

6 Solis, E., Antypa, N., Conijn, J. M., Kelderman, H. & Van der Does, W. Psychometric properties of the Leiden Index of Depression Sensitivity (LEIDS). *Psychol Assess* **29**, 158-171 (2017).
